# Overcoming resistance to BRAF inhibition in BRAF-mutated metastatic melanoma

**DOI:** 10.18632/oncotarget.2602

**Published:** 2014-10-18

**Authors:** Francesco Spagnolo, Paola Ghiorzo, Paola Queirolo

**Affiliations:** ^1^ Department of Plastic and Reconstructive Surgery - IRCCS Azienda Ospedaliera Universitaria San Martino - IST - Istituto Nazionale per la Ricerca sul Cancro - Genova, Italy; ^2^ Genetics of rare cancers, Department of Internal Medicine and Medical Specialties (DiMI), University of Genoa, Genova, Italy; ^3^ IRCCS Azienda Ospedaliera Universitaria San Martino IST, Istituto Nazionale per la Ricerca sul Cancro, Genova, Italy; ^4^ Department of Medical Oncology - IRCCS Azienda Ospedaliera Universitaria San Martino - IST - Istituto Nazionale per la Ricerca sul Cancro - Genova, Italy

**Keywords:** melanoma, BRAF, vemurafenib, dabrafenib, resistance, BRAF inhibitor, MEK inhibitor

## Abstract

Almost 50% of metastatic melanoma patients harbor a BRAF^V600^ mutation andthe introduction of BRAF inhibitors has improved their treatment options. BRAF inhibitors vemurafenib and dabrafenib achieved improved overall survival over chemotherapy and have been approved for the treatment of BRAF-mutated metastatic melanoma. However, most patients develop mechanisms of acquired resistance and about 15% of them do not achieve tumor regression at all, due to intrinsic resistance to therapy. Moreover, early adaptive responses limit the initial efficacy of BRAF inhibition, leading mostly to incomplete responses that may favor the selection of a sub-population of resistant clones and the acquisition of alterations that cause tumor regrowth and progressive disease.

The purpose of this paper is to review the mechanisms of resistance to therapy with BRAF inhibitors and to discuss the strategies to overcome them based on pre-clinical and clinical evidences.

## INTRODUCTION

Almost 50% of metastatic melanoma patients harbor a BRAF^V600^ mutation and the introduction of BRAF inhibitors (BRAFi) has improved their treatment options [1-3]. BRAFi vemurafenib and dabrafenib have been approved for the treatment of BRAF^V600^-mutated metastatic melanoma. In a recent update of the Phase III study of vemurafenib [2], median overall survival (OS) and progression-free survival (PFS) were significantly longer in the vemurafenib group than in the dacarbazine group (OS: 13.6 vs 9.7 months; PFS: 6.9 vs 1.6 months). In the Phase III study of dabrafenib vs dacarbazine [3], median PFS was 6.9 months compared with 2.7 months and also median OS was significantly longer (18.2 vs 15.6 months). However, most patients treated with BRAFi develop mechanisms of acquired resistance and about 15% of them do not achieve tumor regression at all [2,3].

The purpose of this paper is to review the mechanisms of resistance to therapy with BRAFi and to discuss the strategies to overcome them based on pre-clinical and clinical evidence.

### Signaling Pathways in BRAF-mutated Melanoma

#### The RTK-RAF-MEK-ERK signaling cascade (Figure 1)

**Figure 1:**
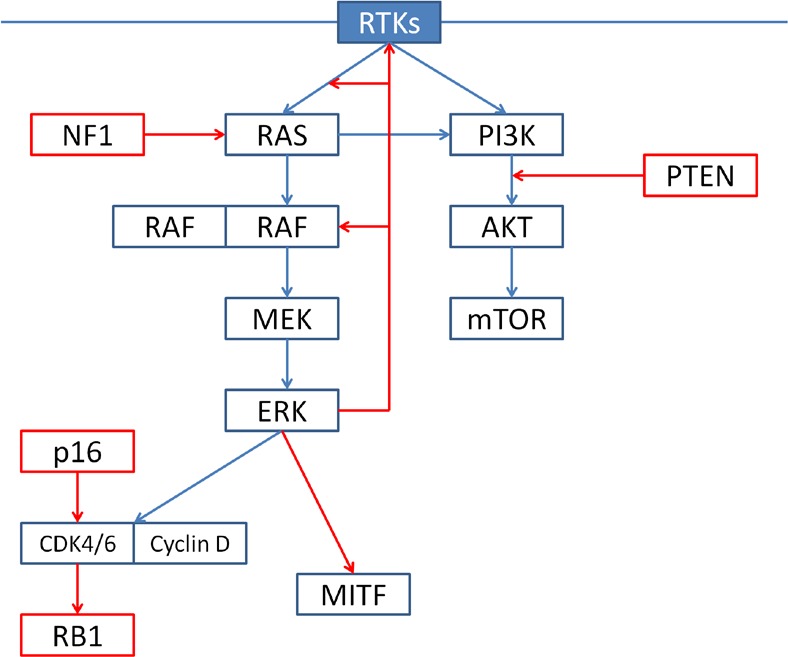
Under physiological conditions, ERK signaling is regulated by extracellular signals binding to receptor tyrosine kinases (RTKs). Activated RTKs promote RAS-mediated dimerization of RAF; wild-type RAF, as hetero- or homodimers, phosphorylate and activate MEK1/2, which in turn phosphorylate and activate ERK1/2. Activated ERK promote cell cycle progression and proliferation and negatively regulates upstream signaling components (negative feedback). RTKs also regulate the PI3K-AKT-mTOR pathway. The two pathways interact at multiple points: most importantly, RAS directly binds and activates PI3K.

The mitogen-activated protein kinase (MAPK) pathway plays an important role in the pathogenesis of melanoma. RAF kinases ARAF, BRAF and CRAF are key components of the pathway, which is physiologically activated when extracellular signals bind to their cognate membrane receptor, typically a receptor tyrosine kinases (RTK). In the absence of such signal, RAF kinases are in an inactive conformation, with the N terminus inhibiting the catalytic C terminus. In the presence of an upstream stimulus, RAS-GTP binds RAF at its N-terminus, relieving its auto-inhibition. The activity of wild-type RAF proteins requires the formation of homo- and heterodimers, which is promoted by RAS activation. In addition, RAF proteins require chaperone or scaffold proteins, such as heat shock protein 90 and the adaptor protein 14-3-3, which stabilize their structure [4].

Activated RAF kinases phosphorylate and activate MEK1/2, which in turn activate ERK1/2. ERK regulates cellular proliferation, survival and differentiation; the activation of ERK is regulated by a complex network of negative-feedback interactions through direct phosphorylation of the components of the RTK-RAS-MAPK cascade by ERK and through the induction of genes which inhibit activation of the pathway, such as Sprouty (SPRY) proteins and dual-specifity phosphatases (DUSPs) [4].

#### BRAF^V600^ mutations (Figure 2)

**Figure 2:**
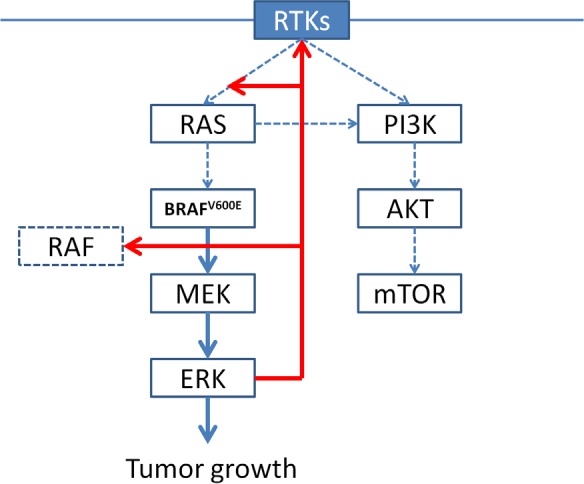
In BRAF-mutated cells, BRAFV600E is constitutively active as a monomer, leading to high ERK signaling and elevated ERK-dependent transcriptional output, including negative-feedback components. As a result, RAS expression is low and does not promote RAF dimerization, and PI3K/AKT signaling is substantially attenuated.

About 50% of melanomas harbor an activating mutation in BRAF, the most common being BRAF^V600E^ [1], which renders the kinase constitutively active. In contrast to wild-type BRAF, the mutated forms of BRAF are active as monomers. In BRAF-mutated melanomas, RAS is negatively regulated by ERK-dependent feedback: BRAF-mutated cells have hyperactive ERK signaling and elevated ERK-dependent transcriptional output, including negative-feedback components. Feedback suppression takes place at multiple levels downstream of RTKs: as a result, RAS expression is low in BRAF-mutated cells and BRAF^V600E^ exists mainly as a monomer, which is not dependent on RAS-GTP induced dimerization [5]. In addition, ERK negative feedback substantially attenuates PI3K/AKT signaling [5].

#### BRAF inhibition

BRAFi vemurafenib inhibits different RAF kinases with different half-maximum inhibitory concentrations: 10 nM for BRAF V600E, 15 nM for CRAF, 35 nM for ARAF and 40 nM for wild-type BRAF [6]. Vemurafenib inhibits ERK signaling only in BRAF-mutated tumors, while wild-type BRAF tumors and, especially, those with RAS mutations show a paradoxical activation of ERK due to the transactivation of RAF dimers [6]. In fact, in wild-type cells, BRAF and CRAF form homo- and heterodimers on RAS activation; vemurafenib binding to one member of the dimer causes an allosteric transactivation of the drug-free protomer and activation of MEK/ERK. The activation of MEK/ERK is enhanced when RAS is overexpressed [6]: this is consistent with the pre-clinical evidence that activated RAS promotes the dimerization of RAF and with the clinical evidence of RAS mutations in most cutaneous squamous cell carcinomas and keratoacanthomas which develop in patients treated with BRAFi [7]. In contrast, the activity of RAS in BRAF-mutated cells is low due to ERK negative-feedback signaling and insufficient to promote RAF dimerization: as a result, RAF exists predominantly as a monomer which responds to selective BRAFi. The relief of ERK negative-feedback during treatment with BRAFi may play a role in the mechanisms of resistance to BRAF inhibition and it will be discussed later.

#### The PI3K/AKT/mTOR pathway (Figure 1)

The PI3K/AKT/mTOR kinase cascade is triggered by RTKs and G-protein-coupled receptors situated at the cell surface. When binding their extracellular ligands, these receptors sequester the regulatory subunit of PI3K, allowing the catalytic subunit to catalyze the phosphorylation of phosphatidylinositol 4,5-bisphosphate (PIP2) to phosphatidylinositol 3,4,5-trisphosphate (PIP3). PIP3 activates downstream signaling components, including the protein kinase AKT, and it is antagonized by the tumor suppressor phosphatase and tensin homolog (PTEN). AKT phosphorylates many survival, proliferation and motility factors, including the tuberous sclerosis protein complex 1 (TSC1) and TSC2, which in turn lead to activation of mTOR complex 1 (mTORC1), a key regulator of cellular growth and protein synthesis [8,9].

#### PI3K/AKT/mTOR and RAS/RAF/MEK/ERK pathway cross-talk

The PI3K/AKT/mTOR and RAS/RAF/MEK/ERK pathways interact at multiple points, resulting in cross-activation, cross-inhibition, and pathway convergence. Most importantly, RAS directly binds and activates PI3K; in addition, ERK phosphorylates TSC2, activating mTORC1. Cross-inhibition between the two pathways may occur under certain circumstances; for example, under high insulin-like growth factor 1 (IGF1) stimulation, AKT can inhibit RAF activity by phosphorylating its regulatory domain [8,9].

### Mechanisms of Resistance

Numerous mechanisms of resistance have been predicted and detected based on *in vitro* and *in vivo* models and many of them have been confirmed on pre- and post-treatment tumor samples (Table 1). Resistant tumors may arise under selective pressure of therapy from pre-existing resistant subclones or as a result of an evolutionary process during treatment, or a combination of both. A detailed understanding of the causes of resistance to BRAFi is necessary to develop more effective treatment strategies. These mechanisms are largely classifiable as either primary/intrinsic, when no clinical benefit is achieved, or secondary/acquired, when progressive disease is observed after a clinical benefit. Moreover, mechanisms of adaptive resistance arise early during exposure to BRAFi and may explain why clinical responses to therapy are mostly partial responses, with complete response rate being in the range of only 3-6% in the Phase III studies [2,3].

**Table 1:** Mechanisms of Resistance to BRAF inhibition

	Intrinsic/Acquired/Adaptive	Pathway	Mechanism of resistance	Frequency (tumor samples)	Reference
**Mutations in NRAS**	Intrinsic/Acquired	MAPK	NRAS activating mutations (NRAS^Q61^, NRAS^T58^, NRAS^G13R^) promote enhanced RAF dimerization; RAF inhibitors binding of one member of the dimer results in allosteric transactivation of the drug-free protomer and activation of MEK/ERK	8-23%	12, 18, 29, 32, 33, 39
**Loss of NF1**	Intrinsic/Acquired	MAPK	Functional inactivation of NF1 leads to activation of the signaling pathways downstream of RAS, including PI3K/AKT and MAPK cascade	2-4%	12, 20, 22-24
**COT expression**	Intrinsic/Acquired	MAPK	COT activates ERK primarily through MEK-dependent mechanisms that do not require RAF signaling	Not available	25
Activating MEK1/2 mutations	Intrinsic/Acquired	MAPK	MEK1 is situated immediately downstream of RAF proteins in the MAPK pathway and promotes ERK phosphorylation; MEK2 forms heterodimers with MEK1 which activate ERK. MEK1^P124S^, MEK1^P124L^, MEK1^I111S^, MEK1^G276W^, MEK1^F53Y^ and MEK1^V154I^ do not seem to confer resistance; MEK1^C121S^, MEK1^Q56P^, MEK1^K57E^, MEK1^E203K^, MEK1^V60E^, MEK1^G128V^ and MEK2^F57C^, MEK2^C125S^, MEK2^V35M^, MEK2^L46F^, MEK2^N126D^ were associated with acquired resistance.	3-15% (only mutations associated with resistance were included)	12, 18, 29, 32, 43-45
**Elevated CRAF**	Intrinsic/Acquired	MAPK	Elevated CRAF protein levels have been associated with increased levels of phosphorylated ERK1/2 levels and may account for the acquisition of resistance to BRAFi due to increased RAF dimerization	Not available	48
**Alternative splicing of V600E BRAF**	Acquired	MAPK	Due to high dimerization property irrespective of RAS status, strongly activates MEK and ERK1/2 in the presence of a RAF inhibitor	13-32%	29, 32, 38
**V600E BRAF copy number amplification**	Acquired	MAPK	MEK/ERK reactivation in a RAS and CRAF-independent manner due to an increased expression of BRAF	8-20%	12, 16, 29, 39, 41
**Relief of ERK negative-feedback**	Adaptive	MAPK	The relief of ERK negative feedback leads to an increased expression of RAS and restores sensitivity to growth factors	Not applicable	4, 5
**Loss of PTEN**	Intrinsic/Acquired	PI3K/AKT	Loss of PTEN leads to AKT activation; melanoma cell lines with PTEN deletion have an impaired apoptotic response due to an inability to up-regulate BIM upon BRAF or MEK inhibition	10-33%	12, 14-16, 18
Alterations of PI3K-AKT pathway	Intrinsic/Acquired/Adaptive	PI3K/AKT	AKT1/3 mutations (Q79K and E17K), mutations in PI3K-AKT positive-regulatory genes (PIK3CA and PIK3CG) and in negative-regulatory genes (PIK3R2 and PHLPP1) up-regulate the PI3K-AKT pathway; the missense mutation AKT1^A102V^ has not been associated with AKT1 activation. Treatment with BRAFi or the combination of BRAF and MEK inhibitor leads to early, adaptive AKT signaling, unleashing a rebound activation of PI3K-AKT pathway.	3-20% (mutations)	12, 29, 32, 49
RTKs Upregulation	Acquired	MAPK and PI3K/AKT	RTK activation can signal either through CRAF or the PI3K pathway	3% (increased levels of IGF-1R)	32-37, 52
Stromal secretion of HGF	Intrinsic	MAPK and PI3K/AK T	HGF activates ERK1/2 through c-MET	Not available	26, 27
RAC1^P29S^ mutations	Intrinsic	Other	RAC1 is a RAS-related GTPase that regulates cell proliferation and migration	7%	12
HOXD8 mutations	Intrinsic	Other	HOXD8 is a homeobox transcription factor that has been shown to be dysregulated in multiple cancers	2%	12
Dysregulatio n of CDK4 and/or cyclin D1	Intrinsic	Other	Cyclin D1 regulates proliferation binding CDK4 and CDK6, which in turn phosphorylate the retinoblastoma protein and lead to progression through the cell cycle	7-24%	16, 19, 29
MITF amplification and relief of MITF suppression	Acquired/Adaptive	Other	MITF encodes a master lineage transcription factor which modulates melanocyte development and promotes cell survival. ERK1/2 pathway inhibition relieves suppression of MITF.	2% (MITF amplification)	12, 53-56
Upregulation of FOXD3	Adaptive	Other	Forkhead box D3 (FOXD3) is a stem cell/pluripotency transcription factor: it was found to be upregulated following MAPK inhibition, conferring resistance to cell death	Not applicable	50-52

### Mechanisms of Primary/Intrinsic Resistance

#### RAC1^P29S^ mutations

RAC1 is a RAS-related GTPase that regulates cell proliferation and migration [10,11]; RAC1^P29S^ is a recurrent UV-signature mutation in cutaneous non-acral melanomas and was the third most frequent activating mutation (9.2%) after those of BRAF and NRAS in a large cohort (n=147) of exome-sequenced melanomas [11]. The RAC1^P29S^ mutation was less frequent, but not mutually exclusive, in NRAS or BRAF mutated melanomas (6.2% vs 12.5%).

Clinical evidence suggests that activating RAC1 mutations may confer resistance to BRAFi: in a cohort of 45 patients treated with BRAFi, among 14 patients exhibiting intrinsic resistance, three pretreatment sample harbored RAC1^P29S^ mutations, and in one of them there was no other alteration known to confer resistance to therapy; no patient who achieved response to therapy harbored this mutation (P=0.026) [12].

RAC1 effectors include various protein kinases, offering a target for pharmacological inhibition, which may be of therapeutic value in the treatment of melanomas harboring the RAC1^P29S^ mutations, although experimental evidence in support of this hypothesis is necessary.

#### Loss of PTEN

PTEN functions as a tumor suppressor by inhibiting PI3K signaling [13]. Loss of PTEN, which is observed in 10-33% of melanoma specimens [14-16], may contribute to intrinsic resistance to BRAFi via increased PI3K/AKT signaling when BRAF is inhibited and suppression of apoptosis mediated by proapoptotic protein BIM, member of the Bcl-2 protein family [14]. It is likely that loss of PTEN alone is not sufficient to confer resistance to BRAFi, but only when it is concurrent with other alterations. In fact, even if AKT activation is sufficient to provide resistance to vemurafenib-mediated apoptosis [17], PTEN loss is not always well correlated with increased AKT activation [14] and responses have been observed even in patients with complete loss of PTEN [12,18]. Nevertheless, cell lines with functional inactivation of PTEN seem to be less sensitive to BRAFi than wild-type PTEN melanoma cells and this was also observed in the clinical setting. In a study by Nathanson et al. [16], patients with wild-type PTEN treated with BRAF inhibitor dabrafenib had longer PFS compared with patients with at least one mutated allele of PTEN (32.1 vs 18.3 weeks; p=0.066), and a modest association between low expression of PTEN and lower response was observed in the phase 2 study of vemurafenib as well [18].

Dual BRAF/PI3K inhibition restores apoptosis in PTEN null cells [14], suggesting that such combination may overcome this mechanism of resistance.

#### Dysregulation of cyclin-dependent kinase 4 (CDK4) and/or cyclin D1

Cyclin D1 regulates proliferation binding both CDK4 and CDK6, which in turn phosphorylate the retinoblastoma protein and lead to progression through the cell cycle. Under physiologic conditions, p16^INK4A^ (encoded by CDKN2A) negatively regulates CDK4 function [19]. Smalley and colleagues [19] found that CDK4 mutations alone did not alter responsiveness to BRAF inhibition, while cyclin D1 overexpression alone increased resistance. Cell lines harboring both a CDK4 mutation and a CCND1 (cyclin D1) amplification were the most resistant to therapy with BRAFi. This is clinically relevant given that CCND1 is amplified in 17% of BRAF^V600E^-mutated metastatic melanoma samples [19]. This pre-clinical model is supported by some clinical evidence: higher copy number of CCND1 (P=0.009) and lower copy number of CDKN2A (P=0.012) at baseline were significantly and independently associated with decreased PFS upon treatment with dabrafenib [16], evidencing the importance of the RB1 (retinoblastoma protein) pathway, which may become a target within a combination approach.

#### Loss of NF1

NF1 functions as a tumor suppressor by inhibiting RAS. Functional inactivation of NF1 leads to activation of the signaling pathways downstream of RAS, including PI3K/AKT and MAPK. Inactivating mutations in NF1 are present in 4% of BRAF-mutated melanomas [20]. NF1 may not only cooperate with BRAF mutations to drive melanomagenesis, but also have a role in the mechanism of primary and secondary resistance to BRAF inhibitors [21-24]. *In vivo* studies suggest that combined MEK and mTOR inhibition [23] and the use of ERK and irreversible RAF inhibitors (such as AZ628) [22] may be strategies to overcome or delay this mechanism of resistance.

#### COT expression

COT activates ERK primarily through MEK-dependent mechanisms that do not require RAF signaling. COT over-expression was identified as a driver of primary and secondary resistance to BRAF inhibition in cell lines and in progressing tumors of patients treated with BRAFi [25].

#### Alterations in RTK signaling (stromal secretion of HGF)

The addition of hepatocyte growth factor (HGF) to BRAF-mutated melanoma cell lines confer resistance to BRAFi [26], hence stromal cells producing large amounts of HGF may be responsible for intrinsic resistance to therapy with BRAFi [27]. This mechanism of resistance is mediated by the activation of HGF receptor c-MET and subsequent activation of both the MAPK and PI3K-AKT signaling pathways and is sensitive, *in vitro* and in a xenograft model, to c-MET and HGF inhibition [26,27]. The combination of a BRAFi with a MEK inhibitor is unlikely to overcome this mechanism of resistance, since the PI3K-AKT pathway is involved as well, whereas the addition of an AKT inhibitor led to the suppression of the majority of HGF-induced resistance *in vitro* [27]. Patients with high baseline HGF serum levels have reduced response rate, PFS and OS [26,27].

#### HOXD8 mutations

HOXD8 is a homeobox transcription factor dysregulated in multiple cancers [12,28]. The detection in a non-responder patient treated with BRAF inhibitors of a nonsense mutation in the HOXD8 gene in the absence of other known resistance-associated alterations suggested that inactivation of this transcription factor may be a cause of intrinsic resistance.

### Mechanisms of Secondary/Acquired Resistance

Most mechanisms of acquired resistance involve a reactivation of the MAPK pathway due to events that can occur upstream, downstream or at the level of BRAF; the PI3K-PTEN-AKT pathway constitutes a second core resistance pathway, which often overlaps with the MAPK pathway. Notably, no gatekeeper mutations have been identified as drivers of acquired resistance. Among 56 progressive tumors samples, deep sequencing of all 18 BRAF exons revealed no BRAF^V600E/K^ secondary mutations and confirmed the persistence of the same BRAF^V600E/K^ mutation in all progressive tumors, demonstrating that BRAFi did not select for minor, preexisting wild-type clones [29]; this was confirmed by another study [30] demonstrating intrapatient homogeneity of BRAF^V600E^ assessed with immunohistochemistry in 171 tumors from 64 patients.

BRAF-mutant melanomas may develop multiple mechanisms of resistance simultaneously, even within a single cell line, and some of them may drive resistance to multiple MAPK inhibitors [31]. In a study on 100 resistant tumor samples from 44 patients [29], an alteration in the MAPK pathway was detected in 70% of the progressive tumors, while alterations of the PI3K –AKT pathway were detected in 22%; in 20% of patients, at least two mechanisms of resistance were detected in the same patient, and the alterations involved both pathways in all cases except for one; 13/16 patients, from whom multiple progressive biopsies were available, harbored multiple mechanisms of resistance. In another study [12], 3/45 patients harbored multiple independent mechanisms of resistance within the same tumor biopsy.

No association was observed between clinical outcome (best response and PFS) and specific mechanisms of resistance [32].

#### Upregulation and activation of the RTKs

Activation of RTKs may drive resistance through the activation of parallel pathways or increasing RAS activity. Upregulation and activation of the platelet-derived growth factor receptor b (PDGFRb) was one of the first alteration identified as an acquired mechanism of resistance to BRAFi treatment [33]. *In vitro*, the PDGFRb cells were insensitive to the PDGFR inhibitor imatinib [34]; however, resistant cells should be sensitive to the combined inhibition of BRAF and the RTK-PI3K-AKT-mTORC pathway [35].

Moreover, immunohistochemistry analysis on 16 pre- and post-treatment samples of metastatic melanoma patients receiving BRAFi (15/16) or MEKi (1/16) revealed that 6 progressing tumor samples acquired EGFR expression. EGFR is not expressed, in general, by melanoma cells, and *in vitro* studies concluded that EGFR expression is disadvantageous for BRAF^V600E^ melanoma cells in the absence of BRAF or MEK inhibitor drugs, but it confers a selective advantage in the presence of these drugs. The addition of an EGFR inhibitor to vemurafenib did not lead to inhibition of cell proliferation, as other drivers of drug resistance, such as RTKs PDGFRB and ERBB3, were implicated. Many RTKs share the RAS-MEK-ERK and the PI3K-AKT signaling pathways: consistent with this, dual inhibition of the two pathways could restore sensitivity to BRAFi in cells with high EGFR expression [36,37]. Acquired EGFR expression is reversed in the absence of the BRAFi, providing evidence that a drug holiday could restore sensitivity when resistance is driven by this mechanism [36].

#### NRAS Mutations

MAPK reactivation due to high levels of activating mutations of NRAS was identified in 2010 by Nazarian and colleagues [33]. This model was validated by the detection of NRAS mutations in 4 of 19 tumor samples with acquired resistance to vemurafenib [38], underlying the importance that this mechanism has in the clinical setting. A combination strategy targeting downstream of NRAS, such as MEK and ERK, and the PI3K/AKT pathway should overcome this resistance mechanism.

#### Alternative splicing of V600E BRAF

Poulikakos et al. [38] detected from *in vitro*-resistant cell lines a 61-kDa form of V600E BRAF (p61BRAF^V600E^), which lacked exons 4–8, a region encoding the RAS-binding domain of BRAF, critical for RAF activation. In cells expressing p61BRAF^V600E^ ERK signaling is resistant to BRAFi. The resistance of p61BRAF^V600E^ to vemurafenib is not due to its inability to bind the inhibitor, but because this variant forms dimers in a RAS-independent manner, strongly activating MEK and ERK in the presence of BRAFi [38]. The use of BRAFi in combination with MEK or ERK inhibitors should delay or prevent this mechanism of resistance, as p61BRAF^V600E^ cells should retain sensitivity to inhibitors of downstream components of the pathway. Nevertheless, an alternative splice variant of BRAF has also been detected in 1/5 patients after treatment with dabrafenib and trametinib in combination [39], raising the notion that this kind of combination may not be effective in this subset of patients. Pre-clinical evidence indicates that PLX7904, a next-generation BRAFi, blocks the growth of vemurafenib-resistant BRAF^V600E^ cells harboring distinct BRAF^V600E^ splice variants [40], so this drug may become a treatment option in this subset of resistant tumors.

#### BRAF^V600E^ copy number amplification

BRAF^V600E^ copy number gain, which results in BRAF^V600E^ over-expression, is sufficient to lead to ERK reactivation in a RAS and CRAF-independent manner [41]. This alteration has been detected in 8-20% of tumor samples after PD with BRAFi [12,16,29,41]. ERK reactivation is saturable with higher doses of BRAFi and is sensitive *in vitro* to MEK inhibition and combined BRAF and MEK inhibition. Nonetheless, BRAF amplification has also been detected in patients after treatment with BRAF and MEK inhibitors in combination [39], pointing out that a combination approach with only these two drugs may not be sufficient to overcome this mechanism of resistance in the clinical setting. Dose-escalation may re-achieve disease control in patients with BRAF copy number gain; since the maximum tolerated dose of dabrafenib has not been determined, with doses up to 300 mg twice daily being tested in a Phase 1 study [42], the feasibility of dose escalation trials with dabrafenib in combination with a MEKi should be assessed in this subset of patients.

#### Activating MEK1/2 mutations

Activating MEK1 and MEK2 mutations may confer resistance in a small percentage of cases [12,18,32,43-45]: a number of different mutations have been identified and only some of them are capable to drive resistance to BRAFi, while the others did not alter sensitivity to BRAFi *in vitro* and did not prevent tumor regressions in the clinical setting (see Table 1 for details). MEK mutations driving resistance to BRAFi may as well confer cross-resistance to MEKi, raising the possibility that BRAF and MEK inhibitors in combination may have limited efficacy in this setting [12,39], while most of these mutations remain sensitive to ERK inhibitors (ERKi) *in vitro* [12,46-47].

#### Elevated CRAF levels

Elevated CRAF protein levels have been associated with increased levels of phosphorylated ERK1/2 levels and may account for the acquisition of resistance to BRAFi due to increased RAF dimerization [48]. Elevated CRAF levels seem to reflect a post-transcriptional regulatory mechanism rather than CRAF gene amplification [48]. In a small subset of BRAF mutated melanoma patients, this alteration may similarly contribute to intrinsic resistance [48].

#### Mutations in PI3K-AKT pathway genes

AKT1/3 mutations and mutations in PI3K-AKT positive-regulatory genes (PIK3CA and PIK3CG) and negative-regulatory genes (PIK3R2 and PHLPP1) may up-regulate the PI3K-AKT pathway [12,29,32], driving resistance to BRAFi.

### Mechanisms of Adaptive Resistance to BRAF inhibition

Early adaptive responses to BRAFi, in addition to limiting the initial efficacy leading to incomplete responses, may favor the selection of a sub-population of resistant clones and the acquisition of alterations that lead to secondary resistance, ultimately causing tumor regrowth and progressive disease.

#### Relief of ERK negative-feedback

In BRAF-mutated melanoma, ERK-dependent feedback suppress RAS activation and BRAF exists predominantly as an active monomer. These cells have decreased sensitivity to growth factors and the transduction of signal from RTKs is suppressed. The exposure to a BRAFi produces a relief of ERK negative feedback, with a consequent enhanced ability of ligands, including growth factors, to activate signaling and an increased expression of RAS-GTP, which promotes the formation of RAF dimers. BRAFi bind to one component of the dimer and cause an allosteric activation of the other one. As a result, the relief of ERK-dependent negative feedback restores sensitivity to growth factors and may diminish the effect of BRAFi through the activation of RAS; however, negative-feedback pathways are partially restored over time, leading to the formation of a new steady state of reactivated ERK signaling, and the level of activation of RAS is variable in different cell lines and, in most melanomas, is not enough to cause resistance in the absence of other activating signals, but can cooperate with mechanisms requiring the presence of active RTK [4]. In addition, it provides a partial explanation to the variability of responses observed in patients treated with BRAFi and it suggests that a combination including an inhibitor of ERK rebound may be necessary to achieve more durable and complete responses.

#### Alterations in the PI3K-PTEN-AKT pathway

The inhibition of the MAPK pathway leads to early, adaptive AKT signaling, unleashing a rebound activation of PI3K-AKT pathway [49]. AKT activation is normally limited by the activity of PTEN [13], supporting the hypothesis that PTEN loss of function has an important role in the failure of BRAFi therapy. This PI3K-AKT-dependent adaptive response to BRAFi, along with the high frequency of melanomas with loss of function of PTEN [14-16] and other alterations linked to this pathway [12,29,32,49], indicate that the addition of a PI3K/AKT inhibitor to MAPK inhibitors may be necessary to obtain a long-term clinical benefit.

#### Upregulation of FOXD3

Forkhead box D3 (FOXD3) is a stem cell/pluripotency transcription factor: it was found to be upregulated following MAPK inhibition, conferring resistance to cell death [50]. The combination of BRAFi with integrin inhibitors should overcome this mechanism of resistance, as the blockade of signals from the extracellular matrix through treatment with integrin inhibitors attenuates the upregulation of FOXD3 *in vitro* [51]. Moreover, since ERBB3 was identified as a direct transcriptional target of FOXD3, a combination with ERBB signaling inhibitors may also have therapeutic value in this setting [52].

#### Upregulation of mitochondrial synthesis and oxidative metabolism

ERK1/2 pathway inhibition relieves suppression of MITF (microphthalmia-associated transcription factor), a promoter for cell survival expressed exclusively in the melanocyte lineage, leading to the upregulation of mitochondrial synthesis and oxidative metabolism through PGC1α (peroxisome proliferator-activated receptor γ coactivator 1α) and promoting survival in the presence of the inhibitor [53-56]. Moreover, amplification of MITF was reported as a mechanism of secondary resistance in a resistant tumor biopsy [12]. Overexpression of MITF desensitizes BRAF-mutated melanoma cells to BRAFi, but MITF expression can be impaired following treatment with histone deacetylase inhibitors (HDACi), suggesting the potential benefit of a combination strategy with MAPK inhibitors. Furthermore, since BRAFi-induced oxidative metabolism renders melanoma cells highly dependent on antioxidant enzymes to survive, the combination with pro-oxidative agents may be a rational strategy to overcome this mechanism of resistance [54].

## DISCUSSION

### Combination versus Sequential strategies

The approval of BRAFi vemurafenib and dabrafenib and MEKi trametinib was a breakthrough in the treatment of metastatic melanoma, even if their efficacy is limited by the development of resistance; these inhibitors now represent key elements to build more effective strategies, such as sequential and combination regimens with other inhibitors and/or immunotherapy.

The understanding of the mechanisms of resistance to BRAFi suggests that stronger anti-tumoral activity may be achieved targeting multiple pathways. The combination of dabrafenib with trametinib significantly increased PFS over dabrafenib alone in a phase II study [57]. Nevertheless, *in vitro* experiments suggest that resistance to BRAFi may confer cross-resistance to MEKi [9,58] and that most mechanisms of resistance involve an activation of additional pathways, such as PI3K/AKT/mTOR, thus the combination of BRAFi only with MEKi, which targets a single pathway, is unlikely to provide long-term disease control. In fact, in the phase III study of dabrafenib+trametinib vs dabrafenib monotherapy, PFS was only slightly better in the combination arm (9.3 vs 8.8 months) (Long, ASCO 2014: Abstract No. 9011). This implies that multiple pathways may be needed to be targeted either in parallel, if not limited by toxicity, or in series, as part of an intermittent dosing schedule.

Resistant tumors, as mentioned earlier, often show an activation of the PI3K/AKT/mTOR pathway; in fact, in addition to the intrinsic and acquired activating alterations, the cross talk between the MAPK and PI3K/AKT pathways results in activation of one pathway when the other is suppressed [8,59], suggesting that a strategy to effectively counteract resistance must rely, at least, on the targeting of both the MAPK/ERK and PI3K/AKT/mTOR pathways. Pre-clinical studies pointed out the synergistic nature of targeting the PI3K/AKT pathway in combination with either BRAF or MEK inhibitors [35,60] and Phase I/II trials are evaluating such combination regimens in patients (Table 2): preliminary data show that these combinations are tolerated and active in patients with BRAF-mutated metastatic melanoma [9].

**Table 2:** Ongoing Phase I-II clinical trials including BRAF-mutated melanoma patients (www.clinicaltrials.gov accessed on 30th May 2014)

NCT	Phase	Drugs	Kind of approach	Sponsor
NCT01897116	I	Vemurafenib (BRAF inhibitor) + Hydroxychloroquine (autophagy inhibitor)	Multi-targeted combination	Abramson Cancer Center (University of Pennsylvania)
NCT01363232	I	BKM120 (PI3K inhibitor) + MEK162 (MEK inhibitor)	Multi-targeted combination	Novartis
NCT01337765	I	BEZ235 (PI3K/mT0R inhibitor) + MEK162	Multi-targeted combination	Novartis
NCTO1390818	I	SAR245409 (PI3K/mT0R inhibitor) + MSC1936369B (MEK inhibitor)	Multi-targeted combination	EMD Serono
NCT01248858	I	GSK2126458 (PI3K inhibitor) + trametinib (MEK inhibitor)	Multi-targeted combination	GlaxoSmithKline
NCT00996892	I	GDC-0941(PI3K inhibitor) + cobimetinib (MEK inhibitor)	Multi-targeted combination	Genentech
NCT00794781	I	E6201 (MEK inhibitor)	Next-generation drug	Eisai Inc.
NCT01657591	I	Vemurafenib + XL888 (HSP90 inhibitor)	Multi-targeted combination	H. Lee Moffitt Cancer Center and Research Institute
NCT01940809	I	Dabrafenib (BRAF inhibitor), trametinib, ipilimumab (anti-CTLA-4 antibody)	Sequential regimen with immunotherapy	National Cancer Institute
NCT02097225	I	AT 13387 (Hsp90 Inhibitor), dabrafenib and trametinib	Multi-targeted combination	National Cancer Institute
NCT01585415	I	Vemurafenib + Adoptive Cell Therapy	Combination with immunotherapy	National Cancer Institute
NCT01835184	I	Vemurafenib + Cabozantinib (multiple tyrosine kinases inhibitor, including RET, MET, and VEGF receptor 2)	Multi-targeted combination	National Cancer Institute
NCT01656642	I	Vemurafenib + MPDL3280A (anti-PDL1 antibody)	Combination with immunotherapy	Genentech
NCT01673737	I	SAR260301 (PI3Kβ-selective inhibitor) +/− Vemurafenib	Multi-targeted combination	Sanofi
NCT01596140	I	Vemurafenib + Everolimus or Temsirolimus (mTOR Inhibitors)	Multi-targeted combination	M.D. Anderson Cancer Center
NCT01767454	I	Dabrafenib +/− trametinib in Combination With ipilimumab	Combination with immunotherapy	GlaxoSmithKline
NCT01512251	I/II	Vemurafenib + BKM120 (PI3K inhibitor)	Multi-targeted combination	University of California, San Francisco
NCT01616199	I/II	Vemurefenib + PX-866 (PI3K inhibitor)	Multi-targeted combination	Oncothyreon Inc.
NCT01902173	I/II	Dabrafenib + GSK2141795 (AKT inhibitor)	Multi-targeted combination	National Cancer Institute
NCT0163 8676	I/II	Vemurafenib + metformin	Multi-targeted combination	James Graham Brown Cancer Center
NCT01841463	I/II	Vemurafenib + P1446A-05 (CDK inhibitor)	Multi-targeted combination	Piramal Enterprises Limited
NCT01943422	I/II	Vemurafenib + High-dose Interferon Alfa-2b	Combination with immunotherapy	John Kirkwood
NCT01959633	I/II	Vemurafenib + PEG-interferon	Combination with immunotherapy	Fondazione Melanoma Onlus
NCT01603212	I/II	Vemurafenib + interleukin-2 + interferon alfa-2b	Combination with immunotherapy	M.D. Anderson Cancer Center
NCT01989585	I/II	Dabrafenib, trametinib, and navitoclax (bcl-2 inhibitor)	Multi-targeted combination	National Cancer Institute
NCT02027961	I/II	MEDI4736 (anti-PDL1) + dabrafenib and trametinib	Combination with immunotherapy	Medlmmune LLC
NCT02130466	I/II	Pembrolizumab (anti-PD1) + dabrafenib and trametinib	Combination with immunotherapy	Merck Sharp & Dohme Corp.
NCT02110355	I/II	AMG232 (MDM2-p53 Inhibitor) + dabrafenib and trametinib	Multi-targeted combination	Amgen
NCT01659151	II	Vemurafenib with lymphodepletion plus adoptive cell transfer and high dose IL-2	Combination with immunotherapy	H. Lee Moffitt Cancer Center and Research Institute
NCT01673854	II	Vemurafenib followed by ipilimumab	Immunotherapy within a re-challenge regimen with vemurafenib	Bristol-Myers Squibb
NCT01519427	II	Selumetinib (MEK inhibitor) + MK2206 (AKT inhibitor)	Multi-targeted combination	National Cancer Institute
NCT01754376	II	Vemurafenib + interleukin-2	Combination with immunotherapy	Massachusetts General Hospital
NCT01983124	II	Vemurafenib + chemotherapy (fotemustine)	Sequential (i.e. after progression with BRAFi) combination	Paola Queirolo
NCT01619774	II	Dabrafenib + trametinib	Sequential (i.e. after progression with BRAFi) combination	M.D. Anderson Cancer Center
NCT01894672	II	LGX818 (BRAF inhibitor)	Alternative dosing schedule	Memorial Sloan-Kettering Cancer Center
NCT01820364	II	LGX818 in rational combination with MEKi or CDK4/6i or FGFRi or PI3Ki or c-Meti	Adaptive sequential (i.e. after progression with BRAFi) combination	Novartis
NCT01495988	II	BRAF inhibitor + Bevacizumab (VEGF inhibitor)	Multi-targeted combination	Melanoma Research Foundation Breakthrough Consortium

ERKi are in early phase clinical trials and may have a value for overcoming resistance mechanisms relying on ERK re-activation. ERKi were more effective than MEKi at overcoming BRAFi resistance conferred by a number of mechanisms (BRAF splice variants, NRAS mutations, MEK mutations, BRAF amplification, RTKs activation) *in vitro* [46,61]; however, ERKi did not overcome resistance mediated by RTK activation in all studies [61], even if a higher level of MAPK suppression was evidenced compared to MEKi. ERKi target wild-type kinases, so they are likely to have a narrow therapeutic index: clinical studies are underway to determine whether ERKi can be delivered at concentrations that are clinically effective (NCT01781429, NCT01358331). Nevertheless, ERKi are likely to have limited efficacy as monotherapy or as sequential therapy after progression with BRAFi; ERK inhibition, in fact, causes the relief of the ERK-dependent negative feedback, leading to activated RAS and PI3K signaling. Pre-clinical evidences support the notion that only a combinatorial strategy targeting both ERK and PI3K/mTOR, if clinically deliverable, may circumvent BRAFi resistance [61].

Anticipating the emergence of multiple mechanism of resistance by targeting multiple pathways in a combinatorial approach may have more success than an adaptive sequential approach. A mathematical model developed by Bozic et al. [62] has shown that concurrent combination therapy is more potent than sequential combination treatment, even when there are no possible mutations that can confer cross-resistance; when there are potential mutations conferring cross-resistance to two or more agents, combination therapy offers some chance while there is no chance with a sequential strategy [62,63]. Although preclinical data suggest that a subset of resistance mechanisms should respond to MEKi, this mathematical model is supported in the clinical setting by the lack of clinical activity of MEKi and BRAFi plus MEKi in patients previously treated with BRAFi. In fact, in the phase II study of MEKi trametinib in BRAF-mutated patients [64], only minimal clinical activity was observed as sequential therapy in patients previously treated with a BRAFi. The lack of clinical activity of MEKi after progression on single agent BRAFi was confirmed in another cohort of patients who were treated after progression with a MEKi or combined BRAF and MEK inhibitors. No objective responses were observed, including patients whose mechanism of resistance would predict a response to downstream inhibition, such as alternative splicing and amplification of BRAF [32]. The limited clinical activity of this sequential regimen is most likely due to intra-patient heterogeneity of resistance [12,29,65-66]*,* cross-resistance [9,58] and alternative pathways activation. Heterogeneous tumor clones probably exist before treatment and cases of tumors developing multiple mechanisms of resistance have been reported [33,65,67]. Intratumor and intrapatient heterogeneity is a challenge for personalized targeted therapies: single tumor samples may lead to underestimation of the tumor genomics landscape and adaptive clinical trials involving the selection of sequential targeted drugs based on the molecular characteristics of a single progressing biopsy are unlikely to provide durable responses [68].

Tumor sampling will have an increasing influence on therapeutic strategies [69]. The analysis of circulating tumor cells or circulating tumor-derived DNA may provide a more accurate and complete genetic profile of patient tumors compared to single tumor biopsies, allowing a better choice of treatments for individual patients [13]. Nevertheless, a highly effective method for the detection and molecular analysis of melanoma circulating cell is yet to be standardized [70,71].

Until the development of more effective diagnostic strategies, the detection of the mechanisms of resistance in clinical practice relies on tumor biopsies. An adaptive sequential strategy using such an approach will be investigated in the clinical setting in a phase II study (NCT01820364) in patients with melanoma who progress on the BRAFi LGX818. Resistant tumors will be biopsied and compared with a pretreatment biopsy to identify the mechanism of resistance. On the basis of the alterations identified in the tumor samples, a second targeted agent from a list of MEK, CDK4/6, FGFR, PI3K, and c-MET inhibitors will be added to the regimen.

### Treatment beyond progression versus drug holiday/re-challenge

Tumor heterogeneity may also explain the apparent contrast between the clinical evidences of continued anti-proliferative activity of BRAFi in BRAF-resistant tumors [72] and the pre-clinical models predicting that in some cases cessation of BRAFi may lead to regression or slower progression of resistant tumors [73-75].

Treatment beyond PD may achieve clinical benefits because sensitive and resistant tumor sub-clones probably coexist, and the discontinuation of treatment would lead to faster progression as a result of the proliferation of sensitive clones [76]. Treatment with BRAFi continued after PD was associated with prolonged OS compared with ceasing treatment in a series of 114 patients treated with BRAFi within Phase I-II-III-IV trials [72]; moreover, in the Phase I study of vemurafenib, 14 patients with isolated PD in a site suitable for local therapy continued treatment beyond progression until systemic progression and, some of them, achieved long-term survival (Kim, Society for Melanoma Research 2012 Congress). These data may be biased by patient selection and future prospective randomized trials are needed to assess the efficacy of treatment beyond progression.

In contrast, apparently, with these observations, Das Thakur et al. demonstrated that some vemurafenib-resistant melanoma cell lines expressing BRAF alternative splicing variants or amplified BRAF may become drug dependent for their proliferation, and that cessation of vemurafenib exposure may lead to regression of these tumors. A discontinuous dosing strategy using either a 4 weeks on/2 weeks off or an individualized regimen, exploiting the fitness disadvantage displayed by drug-resistant cells in the absence of the drug, delayed the onset of drug-resistant disease in two primary human xenograft models. Even if the pause from BRAF inhibition allowed sensitive tumours to re-grow, these cells remained responsive to vemurafenib re-administration. Repeated cycles of vemurafenib treatment using these regimens controlled tumor growth over the course of 7 months of treatment, whereas mice treated on a continuous cycle developed resistance as soon as 2 months after initiation of therapy [73-75]. Other studies seem to support these findings: cells expressing distinct mutant BRAF splice variants grew more efficiently *in vitro* and *in vivo* in the presence rather than in the absence of the vemurafenib analog PLX4720 and, after a drug free interval, became re-sensitized to BRAF inhibition; a drug holiday was also shown to be effective in a melanoma cell line in which resistance was mediated by BRAF copy number amplification [77] and acquired EGFR expression [36]. These findings further provide a rationale to investigate an intermittent regimen with BRAFi: a Phase II clinical study is underway evaluating intermittent dosing of a BRAFi (LGX818) in patients with BRAF-mutated metastatic melanoma (NCT01894672). LGX818 will be administered on a daily schedule dosing for the first 6 weeks; this will be followed by a 2 week break and, thereafter, patients will resume LGX818 on a 2 weeks on/2 weeks off schedule (Table 2).

To what degree these observations made in preclinical models translate into patients is an open issue. In fact, as already mentioned, continued treatment beyond progression with BRAFi seems to be associated with increased survival and no tumor regressions have been reported after treatment discontinuation, suggesting that many and more complicated factors are involved in determining the response of resistant cells to BRAFi in the clinical setting. Nevertheless, anecdotal clinical evidence already exist for the successful application of intermittent dosing or re-challenge of BRAF/MEK inhibitors in melanoma patients. Two patients were successfully rechallenged after progression with vemurafenib or dabrafeib+trametinib [78], and a melanoma patient in which vemurafenib induced proliferation of a previously undetected NRAS-mutant chronic myelomonocytic leukemia was successfully treated using an adjusted intermittent schedule of vemurafenib guided by changes in white cell counts [79].

In clinical practice, until further investigations and availability of new drugs, patients who progress rapidly and in multiple sites during treatment are unlikely to benefit from continuation of MAPK inhibitors and should switch to another line of therapy, while patients with isolated progression, suitable for local treatment, may benefit from continuation of BRAFi treatment, as resistance mechanisms are not always shared by all metastasis [65]. Local treatments may also have a role in case of partial responses with low and accessible tumor burden, when residual lesions, which are a potential evolutionary reservoir of resistant tumor cells, may be removed radically.

### Combinations with immunotherapy

Even if a multi-targeted treatment approach and individualized regimens may improve response rate and their duration, tumor heterogeneity remains a barrier to obtain complete and long-term responses; a combination strategy including the use of an immunotherapeutic agent may provide more durable responses and long-term survival.

There is strong rationale for combined BRAF-targeted therapy and immunotherapy for melanoma. Most studies have supported the idea that BRAFi are unlikely to impair the immune system [80] [81]; on the contrary, these agents may have immunomodulatory properties and enhance immune activation, as treatment with MAPK inhibitors is associated with enhanced expression of melanocytic antigens, antigen recognition by T cells and an influx of cytotoxic T lymphocytes [82] [83-86]. BRAFi paradoxically activate ERK in wild-type cells and this effect was also observed in T cells: BRAFi enhance T cell activation in a concentration-dependent fashion [87]. Moreover, emerging evidence suggests that oncogenic BRAF is immunosuppressive [88], further supporting the rationale for the development of combined targeted therapies with immunotherapy. The first attempt combining BRAFi vemurafenib with anti-CTLA-4 antibody ipilimumab failed due to toxicity issues [89]. Nevetheless, new immunotherapeutic drugs are about to be approved, such as anti-PD-1 antibodies nivolumab and pembrolizumab, which seem to be more tolerated than ipilimumab and, at the same time, to have higher response rates. Increased PD-L1 expression by cancer cells is an escape mechanism from host immunity and reactivation of MAPK seems to be associated with increased expression of PD-L1 [90,91]. Anti-PD-1 agents block the ligation of PD-L1, expressed on cancer and antigen-presenting cells, to PD-1, expressed on T cells, preventing it to suppress T-cell activation and proliferation and to induce T-cell apoptosis. Moreover, tumor expression of PD-L1 is a biomarker of clinical activity of anti-PD-1 agents, further supporting a combination with such drugs. Clinical studies are underway to assess the feasibility and clinical activity of combination therapy with BRAFi, not only with anti-PD-1 and anti-PDL-1 agents, but also with interleukin, adoptive cell therapy and interferon (Table 2). As a matter of fact, the combination of targeted therapy with BRAF inhibitors and interferon has a strong rationale [92]. Interferon receptor subunit IFNAR1 is down-regulated in BRAF-mutated melanoma cells, while BRAF inhibition up-regulates its expression; moreover, vemurafenib and IFNα-2b combination enhances HLA class I antigen expression, which is associated with an increased recognition of melanoma cells by cognate T cells. The combination of vemurafenib and interferon significantly prolonged survival in mice grafted with BRAF-mutated melanoma cells, and two phase I-II studies are evaluating in humans the safety and activity of the combination of vemurafenib plus low-dose peg-interferon (NCT01959633) or high-dose interferon (NCT01943422).

Pre-clinical and clinical evidence suggest that immunotherapy should be incorporated early in the course of MAPK inhibitor treatment, in combination or within an intermittent regimen. In fact, laboratory data demonstrate immune cell infiltration into tumors soon after treatment commencement [82,85] and clinical data available on ipilimumab show that immunotherapy has limited efficacy in almost half patients failing BRAFi treatment [93,94].

## CONCLUSION

The successful development of BRAFi is an example of translation of molecular biology for cancer personalized treatment. However, the benefit provided by BRAFi is limited by resistance and several challenges must be addressed to develop more effective therapeutic strategies. Multiple treatment modalities, including other targeted therapies and immunotherapy, are now available for testing and combination. The understanding of the molecular basis of BRAFi drug resistance may provide insights useful to design the best strategies to prevent or delay the emergence of resistant clones; paradoxically, drug resistance may even be exploited to selectively kill resistant cells [95].

Growing evidence of intra-patient heterogeneity of resistance, cross-resistance and alternative pathways activation highlight the need to perform dedicated molecular analysis of resistance and support the rationale that stronger and longer anti-tumor activity will be obtained when multiple pathways are targeted, either in combination or as part of an intermittent dosing regimen. Moreover, combinatorial and sequential approaches which merge the high response rate and rapidity of tumor regression of BRAFi–based treatments with the long-term responses of immunotherapy may be effective strategies to obtain long-term survival in patients with BRAF-mutated metastatic melanoma, although toxicity issues may arise and should be carefully evaluated in clinical trials.
